# Do patients of integrative anthroposophic pediatric inpatient departments differ? Comparative analysis to all pediatric inpatients in Germany considering demographic and clinical characteristics

**DOI:** 10.1186/s12889-019-7972-x

**Published:** 2019-12-03

**Authors:** Katharina Fetz, Thomas Ostermann, Melanie Schwermer, Sebastian Appelbaum, Jan Vagedes, Tycho Jan Zuzak, Alfred Längler

**Affiliations:** 1 Department of Psychology and Psychotherapy, Chair of Research Methodology and Statistics in Psychology, Witten/Herdecke University, Alfred-Herrhausen-Straße 50, 58448 Witten, Germany; 2 Department of Pediatrics, Gemeinschaftskrankenhaus Herdecke, Gerhard-Kienle-Weg 4, 58313 Herdecke, Germany; 3 ARCIM Institute Academic Research in Complementary and Integrative Medicine, Filderstadt, Germany; 4 Department of Neonatology, University Hospital Tuebingen, Calwerstraße 7, 72076 Tübingen, Germany; 5 Department of Pediatric Oncology and Hematology, University Hospital Essen, Hufelandstr.55, 45147 Essen, Germany; 6 Professorship for Integrative Pediatrics, Institute for Integrative Medicine, Witten/Herdecke University, Alfred-Herrhausen-Straße 50, 58448 Witten, Germany

**Keywords:** Integrative medicine, Pediatrics, Patient characteristics, Catchment area, Epidemiology, Anthroposophic medicine

## Abstract

**Background:**

Integrative medicine (IM) is a patient-centered, evidence-based, therapeutic paradigm which combines conventional and complementary approaches. The use of IM in pediatrics has increased in the past two decades and parents’ demand for it is growing. An IM whole systems approach is anthroposophic medicine. Considering the growing demand for integrative approaches in children, it is relevant from a public health perspective to find out which kind of children use IM in Germany and whether they differ from the entirety of pediatric inpatients in Germany. Moreover, it would be interesting to known, whether these patients are willing to travel a longer distance to gain integrative treatment.

**Methods:**

The present study investigates the standard ward documentation datasets of 29,956 patients of all German integrative anthroposophic pediatric inpatient wards from 2005 to 2016 and compares them systematically to collect data of the entirety of all pediatric inpatient wards in Germany. Apart from patients’ age and gender, and the ICD-10 admission diagnoses, the geographical catchment area of the hospitals were analyzed.

**Results:**

Sociodemographic characteristics of pediatric inpatients in the integrative anthroposophic departments (IAH) did not differ from the entirety of all pediatric inpatients. Regarding clinical characteristics, higher frequencies were found for endocrine, nutritional and metabolic diseases (IAH: 7.24% vs. 2.98%); mental, behavioral, and neurodevelopmental disorders (IAH: 9.83% vs. 3.78%) and nervous diseases (IAH: 8.82% vs. 5.16%) and lower frequencies for general pediatric diseases such as respiratory diseases (IAH: 17.06% vs. 19.83%), digestive diseases (IAH: 3.90% vs. 6.25%), and infectious and parasitic diseases (IAH: 12.88% vs. 14.82%) in comparison to the entirety of all pediatric inpatients in Germany. The IAH showed a broad catchment area, with most patients being from former, Western federal republic of Germany. Large catchment areas (> 100 km) for the IAH are merely covered by severe and chronic diseases.

**Conclusion:**

Pediatric inpatients of IAH do not differ from the entirety of pediatric inpatients in Germany regarding sociodemographic characteristics but show differences regarding clinical characteristics. Parents are willing to travel further distance to get specialized integrative anthroposophic medical care for children with severe and chronic diseases.

## Background

Over the last 20 years, the term “Integrative medicine” has frequently been used in different healthcare sectors and systems to describe health services models that make “use of all appropriate therapeutic and lifestyle approaches, healthcare professionals and disciplines to achieve optimal health and healing” for the patient [1]. As a patient-centered, evidence-based, therapeutic paradigm it combines conventional and complementary approaches to foster patient health and addresses biological, psychosocial, spiritual, and environmental aspects of patients’ wellbeing [2]. Health care professionals of integrative medicine vary depending on the country’s health care system and its national and local regulations but commonly include physicians and non-medical therapists with are “succinct, explicit, and transparent for the integration and subsequent collaboration when treating patients” [3].

In western countries, integrative healthcare approaches have mostly emerged from primary care and, depending on the underlying healthcare system, found their way into secondary and tertiary patient care. In Europe, this has led to specialized hospitals and departments delivering integrative approaches with specializations in natural medicine, homeopathy, anthroposophic medicine or Traditional Chinese medicine [4–11]. In the United States integrative treatment approaches are fostered by the National Center for Complementary and Integrative Health (NCCIH) and have led to the formation of the Consortium of Academic Health Centers for Integrative Medicine [12] which currently includes more than 50 programs and centers.

The clinical use of integrative approaches in pediatrics has increased in the past two decades [2, 4, 6, 13–21]. Integrative medicine is used in children in the US [15, 17, 22–25], Canada [26] and in Europe [4, 6–9, 27, 28]. Integrative medicine for children is provided in private practices, outpatient wards, as well as inpatient wards [8].

Several recent studies report that 30–50% of parents of children with acute or chronic diseases state using integrative medicine for their child [11, 29–31]. The use of integrative medicine seems to be more frequent (> 50%) in children with chronic diseases in the US [22, 32–37]. Factors that are associated with the use of integrative medicine in children are the severity of their disease as well as parents’ use of integrative approaches [14, 38]. It is particularly prominent among affluent and educated parents [39]. A prospective cohort study on holistic pediatric services for inpatients and outpatients in oncology reports nausea, pain, insomnia, and agitation to be the most frequent goals for consultation of integrative medicine in children, as well as questions about herbs, dietary supplements, diet and nutrition, and mind-body therapies, such as guided imagery and biofeedback, and massage [17]. A recent review found a growing establishment of pediatric integrative medicine in academic hospitals in the US and describes pediatric integrative medicine to be a much-needed subspecialty to meet the needs of today’s children [2]. Integrative medicine is especially relevant for pediatric gastroenterology, pain medicine, neurology, oncology, pulmonary and other subspecialties in the US [2]. A very recent study in a large pediatric hospital in the US [19] found that anecdotal and scientific evidence supported the use of integrative approaches in the context of pediatrics.

Two recent publications by Eckert et al. [6] and Anheyer et al. [4] describe the successful implementation of integrative pediatrics at pediatric hospitals in Germany (St Marien, Landshut, Elisabeth hospital Essen) for inpatient and outpatient services. Modalities applied are TCM, relaxation, hypnosis, reflexology, compresses and poultices, aromatherapy, homeopathy, yoga and herbal medicine as well as phytotherapeutic approaches [4]. Optional single-remedy-homeopathy is being integrated into routine pediatric service at the Dr. von Hauner’s Children’s University hospital in Munich [40, 41].

A well-known and frequently used integrative whole systems approach in Germany is anthroposophic medicine [42]. Anthroposophic medicine is based on a holistic understanding of humans and nature and of disease and treatment. The anthroposophic approach is based on a concept of four levels of formative forces and on the model of a three-fold human constitution [42]. It uses medical remedies derived from plants, minerals, and animals, art therapy, eurythmy therapy, and rhythmical massage, counseling, psychotherapy, and specific nursing techniques such as external embrocation [43–45]. Anthroposophic medicine has established therapeutic recommendations for the treatment of children suffering acute gastroenteritis [44], pseudocroup [43], bronchitis [45] and epilepsy. In Germany, there are two pediatric inpatient departments with a distinct focus on integrative anthroposophic medicine, at the Gemeinschaftskrankenhaus Herdecke (community hospital) and the Filderklinik Filderstadt [8, 10]. Even though there is a growing interest of parents on integrative pediatrics, little is known about the inpatient treatment infra-structure, disease burden and patient characteristics.

Until today, most of the available information on pediatric inpatients using integrative medicine is based on qualitative analyses [4, 6], while systematic quantitative analyses of patient characteristics and diagnosis parameters are lacking. Considering the growing demand for integrative approaches and the increasing number of institutions offering and implementing integrative medicine for children [17], it is relevant from a public health perspective to investigate which kind of patients make use of integrative approaches in Germany. In particular, it is of interest whether these patients differ from the entirety of pediatric patients in Germany concerning clinical and demographic characteristics. It has been suggested by previous studies that especially patients with severe [14, 38] and chronic diseases use integrative approaches [22, 32–37] and patients may be willing to travel longer distances to be treated in a hospital with special offers [46] it would be interesting to known, whether these patients are also willing to travel a longer distance to gain integrative treatment, such as anthroposophic medicine. Furthermore, earlier studies have shown that there seems to be a difference between Western Germany and former Eastern Germany considering the use of integrative medicine [47], consequently it would be relevant to know whether there are similar patterns for integrative pediatric patients.

Therefore, the current study aims to investigate patient characteristics and diagnosis parameters of integrative anthroposophic pediatric inpatients and to compare them to data from all pediatric wards in Germany. Our hypotheses were that:

1. Pediatric inpatients treated in these departments do not differ from other German pediatric inpatients concerning demographic characteristics.
2. Pediatric inpatients treated in these departments do not differ from other German pediatric inpatients concerning clinical characteristics.
3. Anthroposophic pediatric inpatient departments have a broad catchment area all over Germany. We hypothesize a higher number of patients from Western Germany in comparison to Eastern Germany. Additionally, we hypothesized that patients with a long travel distance to either one of the hospitals have chronic diseases and patients with a short travel distance predominantly acute disease.

## Methods

### Study design

The current study is based on a secondary data analysis of hospital routine admission and discharge data as reported to the Institute for the Hospital Reimbursement System (InEK) pursuant to paragraph 21 of the German Hospital Remuneration Act. Collection and submission of this data is mandatory for all German hospitals and is defined by the patient’s gender, age, length of hospital stay, postcode area, diagnoses based on the International Classification of Diseases (ICD-10) diagnosis groups, comorbidities and further factors related to the hospitalization. It was conducted according to the Declaration of Helsinki [48] and reported according to the STROBE guidelines [49] for reporting observational cohort studies. A permission from the data security officers of both hospitals for the use of patient data within this study was obtained.

### Setting

In Germany, there are two integrative hospitals focusing on integrative pediatric inpatient care with a focus on anthroposophic medicine: The Gemeinschaftskrankenhaus in Herdecke and the Filderklinik in Filderstadt. Both hospitals treat children with various diseases reaching from general pediatrics to specialized fields as e.g. neonatology, pediatric oncology, and diabetes by means of an integrative approach provided by specialized physicians in integrative pediatrics combining conventional and complementary therapies. The staff includes nurses, pharmacists and therapists trained in integrative medicine. Diagnosis and treatment are delivered in accordance with official pediatric guidelines but include optional treatment of anthroposophic medicine [43, 44] including complementary pharmacotherapy, medicinal baths, rhythmical massages, compresses, and embrocation (rhythmic massages with etheric oils e.g. [50]) as well as art therapy, eurythmy, speech therapies, music therapy [51], and light/ color therapy [52]). Both hospitals are part of the regular medical care and thus funded by the statutory health insurers.

The pediatric ward of the Filderklinik treats on average 1245 patients per year (2005–2016). Apart from general pediatrics, the Filderklinik specifies in epileptology, psychosomatic disorders, neonatology, and cardiology for children. The Filderklinik is an academic teaching hospital of the University of Tubingen.

In the pediatric department of the Gemeinschaftskrankenhaus Herdecke 1750 patients are treated averagely every year (2005–2016). At the department children with a wide spectrum of diseases are being treated; including approaches from diabetology, oncology, neonatology, rheumatology, psychosomatic medicine, and neurology alongside general pediatrics. The Gemeinschaftskrankenhaus is an academic teaching hospital of Witten/Herdecke university.

### Data collection and eligibility criteria

Patient data from both integrative anthroposophic hospitals between 2005 and 2016 were derived from the yearly datasets collected in fulfilment of the German Hospital Remuneration Act. These data are a full sample of all cases and patients giving reasons for admission and discharge with the respective dates. Data were extracted from the hospital documentation system and then transferred into SPSS 24 (IBM). Plausibility checks of all variables were performed prior to data analysis. All data were processed anonymously. Individual patient identification was not possible at any time. Data of the entirety of all pediatric departments in Germany including the anthroposophic hospitals were obtained from the German Federal Statistical Office (Destatis), which collects these data annually in a cumulated form.

Patient characteristics of interest were age, gender and status of insurance. Catchment area was estimated as suggested by Ehara [53]. The distance from patients’ place of residence to either the Gemeinschaftskrankenhaus or the Filderklinik was calculated using Microsoft Excels function “get distance” by means of patients’ zip codes to obtain the catchment area of both hospitals. Additionally, zip codes were used to distinguish between federal states of former Western and Eastern Germany in the analyses. Diagnostic parameters were described in terms of the ICD-10-chapter headings.

### Statistical analysis

All data were analyzed using nonparametric univariate statistics (e.g. Wilcoxon-Test or Chi-Square-Test). Because data from comparable departments were only available as cumulative data, further multivariate analyses were not possible. All statistical analyses were performed using IBM SPSS Version 24.

## Results

### Patient characteristics

The integrative anthroposophic sample includes *N* = 29,956 separate admissions (Gemeinschaftskrankenhaus Herdecke: *n* = 17,503 (58.4%); Filderklinik Filderstadt: *n* = 12,453 (41.6%). The entirety of all pediatric inpatients in Germany consisted of 8,645,173 cases. Patients in the integrative anthroposophic sample were on average 47.6% female showing no difference compared to the percentage of 46.8% of females in the entirety. Age distribution in both samples was right skewed with a median age in the integrative anthroposophic sample of 3 years and a mean age of 5.58 years (SD = 5.90) while in the entirety the median age was 3.5 years with a mean of 5.63 years. (SD = 5.81). Wilcoxon test revealed no statistically significant mean difference concerning mean age between the integrative anthroposophic and the entirety sample (*z* = − 1.49; *p* = .14).

### Diagnostic parameters

#### ICD-diagnoses

Most frequent ICD-chapters in the integrative anthroposophic sample (IAH) were diseases of the respiratory system (*n* = 5019; 17.06%), certain infectious and parasitic diseases (*n* = 3859; 12.88%), and certain conditions originating in the perinatal period (*n* = 3547; 11.84%). In the entirety of all pediatric inpatient wards, most frequent diseases were diseases of the respiratory system (*n* = 1,714,182; 19.83%) and certain infectious and parasitic diseases (*n* = 1,281,000; 14.82%), and injury, poisoning and certain other consequences of external causes (*n* = 999,233; 11.56%).

Higher frequencies were found for endocrine, nutritional and metabolic diseases (IAH: 7.24% vs. 2.98%); mental, behavioral and neurodevelopmental disorders (IAH: 9.83% vs. 3.78%) and diseases of the nervous system (IAH: 8.82% vs. 5.16%) and lower frequencies for general pediatric diseases such as respiratory diseases (IAH: 17.06% vs. 19.83%), diseases of the digestive system (IAH: 3.90% vs. 6.25%), and infectious and parasitic diseases (IAH: 12.88% vs. 14.82%) in comparison to the entirety of all pediatric inpatients in Germany.

An overview of the frequencies and percentages of all ICD-10-chapters in the integrative anthroposophic pediatric hospitals and the entirety of all pediatric hospitals in Germany is provided in Table 1.

**Table 1:** Frequencies of ICD chapters in the integrative anthroposophic pediatric inpatient departments and in the entirety of all pediatric inpatient departments

ICD-10 chapter		integrative anthroposophic pediatric hospitals		entirety of allpediatric hospitals	
		n	%	n	%
A00 - B99	Certain infectious and parasitic diseases	3859	12.88%	1,281,000	14.82%
C00 - D48	Neoplasms	1140	3.81%	264,457	3.06%
D50 - D90	Diseases of the blood and blood-forming organs and certain disorders involving the immune mechanism	277	0.92%	107,363	1.24%
E00 - E90	Endocrine, nutritional and metabolic diseases	2168	7.24%	257,599	2.98%
F00 - F99	Mental, behavioral and neurodevelopmental disorders	2946	9.83%	327,029	3.78%
G00 - G99	Diseases of the nervous system	2642	8.82%	445,815	5.16%
H00 - H59	Diseases of the eye and adnexa	68	0.23%	45,996	0.53%
H60 - H95	Diseases of the ear and mastoid process	172	0.57%	76,118	0.88%
I00 - I99	Diseases of the circulatory system	200	0.67%	116,836	1.35%
J00 - J99	Diseases of the respiratory system	5109	17.06%	1,714,182	19.83%
K00 - K93	Diseases of the digestive system	1168	3.90%	540,752	6.25%
L00 - L99	Diseases of the skin and subcutaneous tissue	499	1.67%	156,706	1.81%
M00 - M99	Diseases of the musculoskeletal system and connective tissue	433	1.45%	174,555	2.02%
N00 - N99	Diseases of the genitourinary system	588	1.96%	247,691	2.87%
O00 - O99	Pregnancy, childbirth and the puerperium	8	0.03%	970	0.01%
P00 - P99	Certain conditions originating in the perinatal period	3547	11.84%	640,136	7.40%
Q00 - Q99	Congenital malformations, deformations and chromosomal abnormalities	473	1.58%	168,625	1.95%
R00 - R99	Symptoms, signs and abnormal clinical and laboratory findings, not elsewhere classified	1550	5.17%	840,874	9.73%
S00 - T98	Injury, poisoning and certain other consequences of external causes	2950	9.85%	999,233	11.56%
Zoo - Z99	External causes of morbidity	0	0.00%	239,055	2.77%
U00 - U99	Factors influencing health status and contact with health services	159	0.53%	29	0.00%
N		29,956	100.00%	8,645,021	100.00%

#### Catchment area

**Fig. 1:**
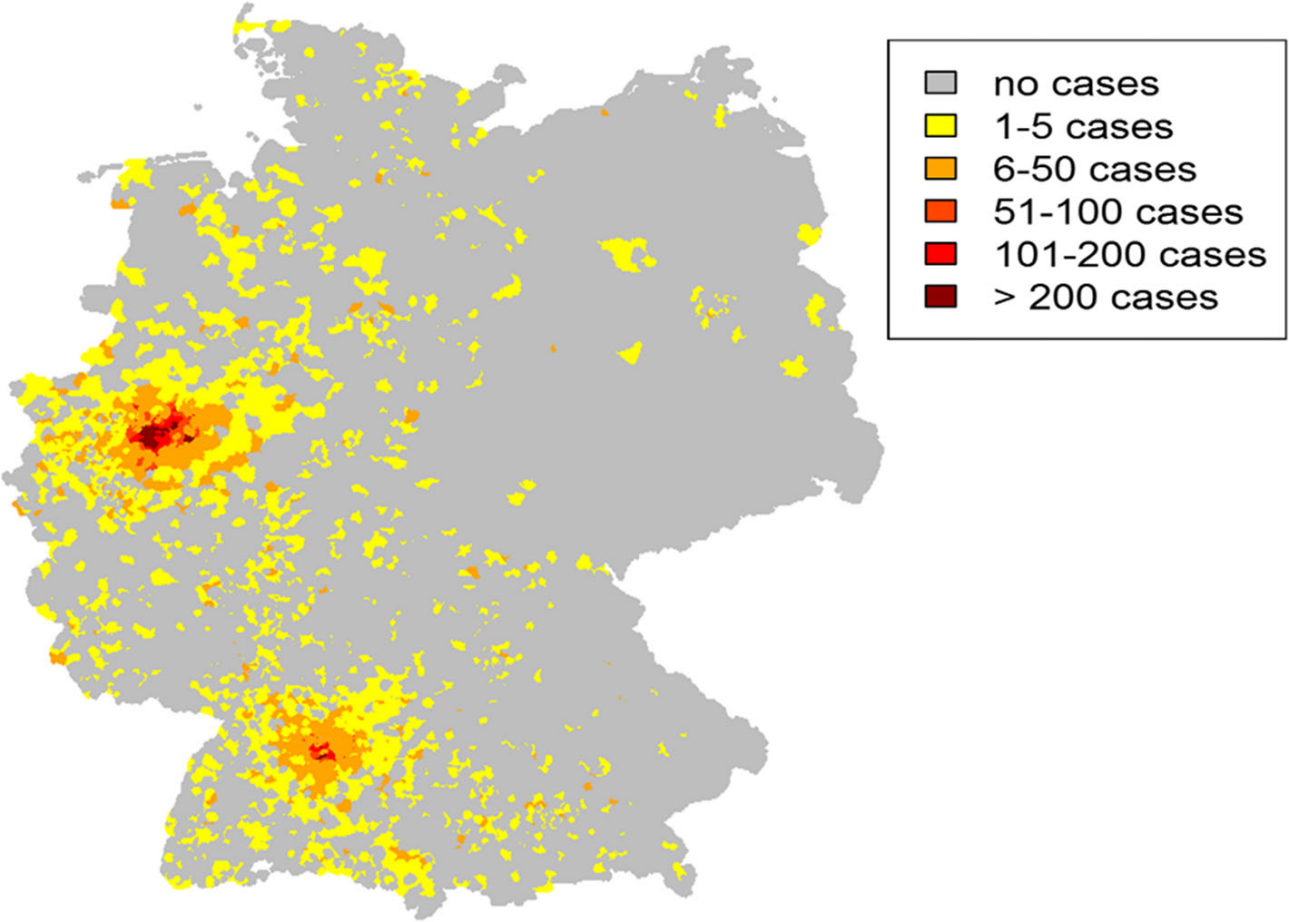
Catchment area of the integrative anthroposophic pediatric inpatient departments in Germany. This heatmap was created based on patients’ postal code data using R Statistical Computing

The catchment areas of the integrative anthroposophic pediatric inpatient wards are illustrated in Fig. 1. The mean distance from patients’ place of residence to one of the hospitals was 61.84 km (SD = 170.27 km). Almost one third (9673 patients; 32.40%) came from the very local catchment area (< 10 km), 13,949 (46.80%) came from regional catchment areas (10–50 km), 2573 (8.6%) came from an extended catchment area between 50 and 100 km and 3614 (12.1%) came from a supra-regional catchment area (> 100 km). A significant difference in the number of patients was observed between former Western Germany (*n* = 29,590; 99.2%) and former Eastern Germany (*n* = 250; 0.8%).

The catchment area groups for the ICD-10 chapters are shown in detail in Table 2. Chi-Square test revealed significant differences between the ICD-10 groups concerning group of catchment area (Chi^2^ = 5765.28; *p* < .001). Causes like pregnancy, childbirth and the puerperium, injuries & poisonings, and diseases of the circulatory system were more locally centered around the hospitals, while diseases of the nervous system, musculoskeletal disorders and neoplasms were reason for patients from more outlying areas to choose one of these hospitals.

**Table 2:** Catchment area and ICD-10 chapters of the integrative anthroposophic pediatric inpatient departments

ICD-10 chapter		catchment area									
		local< 10 km		regional10–50 km		extended50–100 km		supraregional> 100 km		overall	
Certain infectious and parasitic diseases		1578	16%	1808	13%	223	9%	239	7%	3848	13%
Neoplasms		169	2%	495	4%	167	6%	294	8%	1125	4%
Diseases of the blood and blood-forming organs and certain disorders involving the immune mechanism		66	1%	145	1%	39	2%	27	1%	277	1%
Endocrine, nutritional and metabolic diseases		359	4%	1173	8%	353	14%	275	8%	2160	7%
Mental, behavioral and neurodevelopmental disorders		562	6%	1330	10%	434	17%	605	17%	2931	10%
Diseases of the nervous system		408	4%	845	6%	272	11%	1050	29%	2575	9%
Diseases of the eye and adnexa		19	0%	39	0%	7	0%	3	0%	68	0%
Diseases of the ear and mastoid process		56	1%	94	1%	11	0%	11	0%	172	1%
Diseases of the circulatory system		98	1%	77	1%	10	0%	15	0%	200	1%
Diseases of the respiratory system		1965	20%	2541	18%	354	14%	240	7%	5100	17%
Diseases of the digestive system		394	4%	538	4%	105	4%	129	4%	1166	4%
Diseases of the skin and subcutaneous tissue		125	1%	234	2%	61	2%	78	2%	498	2%
Diseases of the musculoskeletal system and connective tissue		70	1%	162	1%	37	1%	163	5%	432	1%
Diseases of the genitourinary system		215	2%	292	2%	35	1%	44	1%	586	2%
Pregnancy, childbirth and the puerperium		0	0%	8	0%	0	0%	0	0%	8	0%
Certain conditions originating in the perinatal period		979	10%	2177	16%	232	9%	152	4%	3540	12%
Congenital malformations, deformations and chromosomal abnormalities		128	1%	246	2%	42	2%	56	2%	472	2%
Symptoms, signs and abnormal clinical and laboratory findings, not elsewhere classified		641	7%	682	5%	119	5%	104	3%	1546	5%
Injury, poisoning and certain other consequences of external causes		1768	18%	1009	7%	64	2%	105	3%	2946	10%
Factors influencing health status and contact with health services		73	1%	54	0%	8	0%	24	1%	159	1%
overall		9673		13,949		2573		3614		29,809	

## Discussion

The aim of our study was to investigate patient characteristics and diagnosis parameters of integrative anthroposophic pediatric inpatients and compare them to data from all pediatric wards in Germany. We found no difference between pediatric patients in the integrative anthroposophic hospitals and the entirety of all pediatric hospitals concerning age and gender distribution. We furthermore hypothesized that patients treated in these department do not differ from the entirety considering clinical characteristics, such as the frequency of the ICD-10 diagnoses. Our findings did not support this hypothesis; in fact, our data indicated that the situation is much more complex. While there are disease categories that show great similarities in their frequencies compared to the entirety of all pediatric departments in Germany such as diseases of the skin and subcutaneous tissue, congenital malformations and deformations and chromosomal abnormalities, we found comparatively higher frequencies for perinatal, neurological and behavioral diseases. Moreover, we found lower frequencies for diseases from the field of general pediatrics such as digestive diseases, respiratory diseases, and infectious and parasitic diseases. Our data showed a broad catchment area for the integrative anthroposophic pediatric hospitals with a significantly higher number of patients from the former Western part of Germany compared to the former Eastern part. We were furthermore able to demonstrate, that patients with a long travel distance to either one of the integrative anthroposophic hospitals have chronic diseases and patients with a short travel distance predominantly acute disease.

### Comparison to previous findings

In line with former research our data suggests that integrative medicine for children is used for acute as well as chronic diseases [11, 29–31]. Our results showed a higher number of pediatric inpatients with endocrine, nutritional and metabolic diseases, mental, behavioral and neurodevelopmental diseases, nervous diseases and perinatal diseases in comparison to the entirety of pediatric inpatient departments in Germany. This circumstance may most likely be due to the certain specializations of the integrative anthroposophic hospitals. However, these diseases also tend to be severe and chronic diseases. In line with this, earlier research showed the use of integrative medicine to be more frequent in children with severe and chronic diseases [14, 22, 32–38].

In our study, the most frequent diseases in the integrative anthroposophic pediatric inpatient departments were respiratory diseases, infectious and parasitic diseases, and perinatal diseases. In contrast, other studies on integrative pediatric in- and outpatient service found nausea, pain, insomnia, and agitation to be the most frequent consultation indications [17]. This difference is most likely due to the oncological setting of this study, which shows a limited comparability to our study setting and patients. In line with a current review [2] we found suggestions that integrative medicine for children is a much-needed subspecialty to meet the need of today’s children and that it shows specific relevance for certain disciplines. In line with previous research [47] we found a larger number of patients using integrative pediatrics from former Western Germany in comparison to former Eastern Germany.

While most of the ICD-10 categories had a local and regional catchment area, the supra-regional catchment area (> 100 km) seems to be merely covered by children treated for diseases of the nervous system, neoplasms and diseases of the musculoskeletal system and connective tissue. This circumstance is not surprising as these diseases are part of the specialties of both hospitals. In accordance with findings of (Adams 1991) it seems plausible that parents of patients with these diseases take a longer travel time to the hospital to get treated in one of the specialized integrative anthroposophic hospitals. It is in line with earlier research reporting that parents of children with chronic and severe diseases use integrative medicine more frequently [22, 32–37]. In line with our results, a recent study [54] found a large catchment area (138 km) for children with chronic pain treated in a specialized pediatric chronic pain ward in Germany. This result was interpreted as an indicator for inadequate resources in other regions. However, in other studies larger catchment areas were also found in hospitals specialized i.e. in knee surgery [55]. Thus, a broad catchment area can also be interpreted as an indicator of quality of care. Currently, there is a trend towards a centralization of specific healthcare supply in particular in the hospital sector in the German healthcare system [56]. There seems to be a comparable pattern within integrative anthroposophic pediatrics as a highly specialized treatment approach.

### Comparison to other integrative services in Germany

The present study investigated patients of two specialized integrative pediatric inpatient departments with a distinct focus on anthroposophic medicine. While these departments are not the only institutions offering integrative approaches for children there are some differences in the kind of integrative healthcare service offered: The Elisabeth hospital in Essen [4] and St Marien hospital in Landshut [6] offer consultancy for pediatric inpatients and outpatients. In contrast to this approach, integrative anthroposophic hospitals have fully implemented integrative approaches into the daily clinical routine for all patients admitted.

### Strengths and limitations

With our study we aimed to contribute to the better understanding of patient populations using pediatric integrative medicine in Germany. To our knowledge, this is the first systematic comparison of a large sample of integrative pediatric patients to the entirety of pediatric inpatients in Germany. A significant limitation of our study is that in consequence of being a secondary data analysis some subgroup comparisons were not possible, because we were only able to get cumulated data from the German Federal Statistical Office. In particular, there were no data available concerning the catchment areas of other pediatric inpatient departments to compare our data to.

## Conclusions

In line with our hypothesis, pediatric inpatients of integrative anthroposophic inpatient departments do not differ from the entirety of pediatric inpatients in Germany with regard to sociodemographic characteristics. Considering clinical characteristics, the situation seems to be more complex and patterns of similarities and differences are heterogenous. The integrative anthroposophic pediatric departments show a broad catchment area all over Germany, with a majority of patients being from former Western Germany. Larger catchment areas for the integrative anthroposophic pediatric hospitals are merely covered by severe and chronic diseases.

## Data Availability

Raw data are available only for analysis purposes and only to dedicated staff of our research group. As the original data is patient hospital data we have no permission to share it.

## Abbreviations

Destatis: German Federal Statistical Office
IAH: Integrative anthroposophic pediatric hospitals
ICD: International Classification of Diseases
IM: Integrative Medicine
InEK: Institute for the Hospital Reimbursement System
M: Mean
N: Sample size
NCCIH: National Center for Complementary and Integrative Health
SD: Standard deviation
SPSS: Statistical Package for the Social Sciences
STROBE: Strengthening the Reporting of Observational Studies in Epidemiology
